# Genome Sequences of Nine Vesicular Stomatitis Virus Isolates from South America

**DOI:** 10.1128/genomeA.00249-16

**Published:** 2016-04-14

**Authors:** Veronica L. Fowler, David J. King, Emma L. A. Howson, Mikidache Madi, Steven J. Pauszek, Luis L. Rodriguez, Nick J. Knowles, Valérie Mioulet, Donald P. King

**Affiliations:** a The Pirbright Institute, Pirbright, Woking, Surrey, United Kingdom; b Plum Island Animal Disease Center, Orient, New York, USA

## Abstract

We report nine full-genome sequences of vesicular stomatitis virus obtained by Illumina next-generation sequencing of RNA, isolated from either cattle epithelial suspensions or cell culture supernatants. Seven of these viral genomes belonged to the New Jersey serotype/species (clade III), while two isolates belonged to the Indiana serotype/species.

## GENOME ANNOUNCEMENT

The genus *Vesiculovirus* (order *Mononegavirales*, family *Rhabdoviridae*) consists of nine virus species, four of which may cause vesicular stomatitis (VS), vesicular stomatitis New Jersey virus (VSNJV), vesicular stomatitis Indiana virus (VSIV), vesicular stomatitis Alagoas virus (VSAV), and Cocal virus (COCV)*.* VS is endemic in southern Mexico, Central America, and the northern part of South America and mainly affects horses and cattle (1). Occasionally, pigs, sheep, goats, llamas, and alpacas may also be affected (1). VSV is a single-stranded negative-sense RNA virus of approximately 11 kb in length that encodes five proteins: nucleocapsid (N), phosphoprotein (P), matrix (M), glycoprotein (G), and polymerase (L) (2).

In this study, nine VS viruses were subjected to full-genome sequencing to be used for validation of new molecular diagnostic tests (Fowler et al., unpublished data). RNA was extracted using QIAamp spin column purification, and genomic DNA was depleted using DNase (Life technologies). First- and second-strand synthesis and DNA library generation was performed as previously described (3) but substituting a VSV-gene junction primer (5′ AAA CTA ACA GAT ATC ATG GAC AG 3′) for the first-strand synthesis stage. Consensus sequences were generated using *de novo* assembly; trimmed FASTQ files were processed using IDBA_UD version 1.1.1, with an optimum *k*-mer length determined within the program (4). The identity of the contigs produced was confirmed via a BLAST search, and, if required, contigs were assembled manually using BioEdit version 7.2.5 (5). Using the *de novo* output as a reference, alignments were performed using BWA-MEM, SAM/BAM processing was performed using SAMtools (6), coverage plots were generated using BEDTools (7), and alignments were visually checked using Tablet (8). Final consensus sequences were generated using SAMtools mpileup. The total genome recovered for the seven New Jersey isolates ranged from 10,957 to 11,118 nucleotides in length with a mean coverage of 95.24 reads/genome position, obtaining 99.50% of the genomes. For these isolates, the total number of missing nucleotides are located in noncoding regions (*n* = 177), N (*n* = 19; Ecuador/85 *n* = 1, COL/1/93 *n* = 4, 29453/COL/00 *n* = 14), M (*n* = 1; 27405/COL/98), and L (*n* = 64; COL/1/93 *n* = 36, 27775/COL/98 *n* = 1, 27405/COL/98 *n* = 27). These isolates grouped into VSNJV clade III, which contains all previously reported VSNJVs from South America. The total genome recovered for the two VSIV isolates (from serotype Indiana) ranged from 11,099 to 11,134 nucleotides in length with a mean coverage of 339.46 reads/genome site, obtaining 99.67% of the genomes. For these isolates, the total number of missing nucleotides are located in the noncoding region (*n* = 99) and L (*n* = 2; 29705/COL/01).

### Nucleotide sequence accession numbers.

The complete genome sequences for these isolates have been deposited in the GenBank database under the accession numbers KU296051 to KU296059.
